# Sex and age bias viral burden and interferon responses during SARS-CoV-2 infection in ferrets

**DOI:** 10.1038/s41598-021-93855-9

**Published:** 2021-07-15

**Authors:** Magen E. Francis, Brian Richardson, Una Goncin, Mara McNeil, Melissa Rioux, Mary K. Foley, Anni Ge, Roger D. Pechous, Jason Kindrachuk, Cheryl M. Cameron, Christopher Richardson, Jocelyne Lew, Steven Machtaler, Mark J. Cameron, Volker Gerdts, Darryl Falzarano, Alyson A. Kelvin

**Affiliations:** 1 Department of Microbiology and Immunology, Faculty of Medicine, Dalhousie University, Halifax, NS B3H 4R2 Canada; 2 Vaccine and Infectious Disease Organization-International Vaccine Centre (VIDO-InterVac), University of Saskatchewan, Saskatoon, SK S7N 5E3 Canada; 3 Department of Population and Quantitative Health Sciences, Case Western Reserve University, Cleveland, OH 44106 USA; 4 Department of Medical Imaging, University of Saskatchewan, Saskatoon, SK S7N 0W8 Canada; 5 Department of Microbiology and Immunology, University of Arkansas for Medical Sciences, Little Rock, AK 72205 USA; 6 Laboratory of Emerging and Re-emerging Viruses, Department of Medical Microbiology, University of Manitoba, Winnipeg, MB R3E 0J9 Canada; 7 Department of Nutrition, Case Western Reserve University, Cleveland, OH 44106 USA; 8 Department of Veterinary Microbiology, Western College of Veterinary Medicine, University of Saskatchewan, Saskatoon, SK S7N 5B4 Canada; 9 Department of Pediatrics, Division of Infectious Disease, Faculty of Medicine, Dalhousie University, Halifax, NS B3K 6R8 Canada; 10 Canadian Centre for Vaccinology, IWK Health Centre, 5980 University Ave, 4th Floor, R4020, Halifax, NS B3K 6R8 Canada

**Keywords:** Virology, SARS-CoV-2, Viral host response, Viral immune evasion, Viral pathogenesis, Virus-host interactions, Infection, Immunology, Viral infection

## Abstract

SARS-CoV-2 (Severe Acute Respiratory Syndrome Coronavirus 2) hospitalizations and deaths disportionally affect males and older ages. Here we investigated the impact of male sex and age comparing sex-matched or age-matched ferrets infected with SARS-CoV-2. Differences in temperature regulation was identified for male ferrets which was accompanied by prolonged viral replication in the upper respiratory tract after infection. Gene expression analysis of the nasal turbinates indicated that 1-year-old female ferrets had significant increases in interferon response genes post infection which were delayed in males. These results provide insight into COVID-19 and suggests that older males may play a role in viral transmission due to decreased antiviral responses.

## Introduction

In December 2019, Severe Acute Respiratory Syndrome Coronavirus 2 (SARS-CoV-2) emerged in Wuhan, Hubei, China, leading to a pandemic spread where as of April 20, 2021, more than 140 million cases and over 3 million deaths have been confirmed^1,2^. SARS-CoV-2, identified as the causative agent of COVID-19 (Coronavirus disease 2019), is a beta-coronavirus belonging to the *Coronaviridae* family of enveloped positive sense, single-stranded RNA viruses with significant similarities to SARS-CoV-1 which emerged in 2002^3,4^. Infection in humans with SARS-CoV-2 can lead to severe disease, hospitalization, and death in some cases while others may remain subclinical or develop only mild disease; therefore, the clinical picture of COVID-19 is significantly varied and disease can range from mild nasal congestion and sore throat to severe pneumonia with diffuse alveolar damage leading to multi-organ failure and death^5–7^. Non-respiratory symptoms such as loss of taste and smell, the development of microblood clots, strokes, neurological impairment, and long-term complications (Post-Acute Sequelae of COVID-19 (PASC)) have also been reported or associated with infection^8,9^. Epidemiological analysis across several countries has indicated that there are several host factors including sex, age, and co-morbidities that can increase COVID-19 severity. Demographic analysis has inidicated that men have increased severe disease and mortality following SARS-CoV-2 infection compared to women^10,11^. Age also has a clear impact on COVID-19 where case fatality rates (CFR) significantly increase with age^5,11^. Indeed, the CFR for COVID-19 in individuals < 40 years of age is less than 0.2% whereas those aged between 60 and 69, 70–79, and 80+ have CFRs of 3.6%, 8.0%, and + 14%, respectively^11,12^. Thus people > 65 years of age are considered the highest risk group for developing severe illness by the US Centers for Disease Control and Prevention (CDC)^13^. In the US alone, 80% of COVID-19-related fatalities have occurred in patients > 65 years of age^5^ and long-term care facilities have been demonstrated to be particularly vulnerable to COVID-19^14–16^. Clinical identification of COVID-19 in the elderly is also constrained by differences in disease symptoms including tachypnea, unexplained tachycardia and increased blood pressure, altered mental status, muscle pain, and fatigue^14,17^. Understanding COVID-19 disease mechanisms in higher-risk groups, including males and the elderly, remains a significant public health priority and will inform supportive care and treatment modalities as well as vaccine strategies for high-risk groups.

Preclinical models are essential for advancing countermeasures for infectious diseases to human clinical trials. Animal models are indispensable for responses to newly emerging infectious diseases given their importance for preclinical evaluations of vaccines and therapeutics and corresponding insights into disease pathophysiology^18^. However, development of these models requires identification of species that are susceptible to infection as well as recapitulation of human clinical disease. While mice have been traditionally employed in preclinical investigations due to reagent availability and cost, they are not typically susceptible to human clinical viral isolates without adaptation. In contrast, ferrets are commonly susceptible to human viruses, including ebolaviruses, influenza viruses and coronaviruses, and have been used for virus characterization and vaccine testing^18–20^. Additionally, the respiratory tract of ferrets has human-like physiology including similarities in the numbers of terminal branches in the lower respiratory tract, distribution of cellular receptors for viruses, and body:lung surface area ratio^21^. Ferrets display similar symptoms and clinical features to humans during respiratory infections, including fever, nasal discharge, coughing, and weight loss^19,22–25^. They have also been used to investigate age-related host responses, disease severity, and pathogenic immune mechanisms during respiratory virus infection^23,24^. Further, the utility of ferrets for infection studies has been demonstrated in SARS-CoV investigations for vaccine development and innate immune responses, including interferon stimulated genes^19,22,26–31^. Currently, ferrets, domesticated cats, non-human primates, and Syrian hamsters have been shown to be susceptible to infection and support viral replication for preclinical analysis^32–37^. Although not naturally susceptible, genetically modified mice as well as mouse adapted SARS-CoV-2 viruses in wild type mice have also been used for SARS-CoV-2 preclinical modeling^38–40^. Ferret infection studies have shown that the virus can be readily transmitted between animals by contact as well as aerosol transmission^33,41^. Ferrets are sexually dimorphic animals that display sex-biased health conditions suggesting their utility for COVID-19 sex biases^21^. Moreover, age-related viral disease severity has been shown in ferrets by our group and others^23,24,42–45^.

As COVID-19 severity has been associated with the male sex and age, we investigated these biological variables and their contribution to disease in ferrets infected with SARS-CoV-2. Ferrets were chosen for our analysis since we have previous experience showing sex-effects and age-effects in regard to respiratory viral infection in this model^24,43,46^. We characterized sex- and age-related viral load and shedding as well as immune gene expression after SARS-CoV-2 infection. Taken together, our data demonstrated that older males (2-year-old males) had a longer duration of viral shedding from the upper respiratory tract with a delayed induction of interferon stimulated genes compared to 1-year-old females and males. These findings may suggest that antiviral treatment may be more beneficial to older male patients. In addition, our findings highlight that not only sociological and societal factors, but also biological factors including sex hormones, sex chromosomes, and aging, contribute to COVID-19 sex-biases.

## Results

### Minimal clinical differences were observed between groups after SARS-CoV-2 infection

Significant sex and age biases have been observed for severe COVID-19^47–50^. Understanding the molecular mechanisms contributing to these biases is essential for therapeutic development and preventative measures which will provide a greater protection for vulnerable populations such as the elderly and males. Ferrets are often used as a preclinical model for dissecting the molecular mechanisms of human viral diseases including those with sex and age biases^42,51^. Here, we employed ferrets to gain a better understanding of human SARS-CoV-2 infection and the increased risk of severe COVID-19 in 2-year-olds. Ferrets are typically purchased for scientific studies after they have been fixed (i.e., spayed or neutered). To address our questions surrounding the roles of sex and age in severe COVID-19 disease, we acquired intact female and male (adult and aged) ferrets from Triple F farms for our studies. Ferrets can be considered senior at 3 years of age where they can start showing signs of aging such as hair loss, gastrointestinal issues, and increased prevalence of neoplasia^52^. Domesticated ferrets can live over 10 years however, those in the wild typically do not live as long. We have previously seen immunological decline in 2–4 year old ferrets^24^ and even greater immunological differences in extreme aged ferrets 5.5–7 years of age^42^. Here we sought out to investigate the roles of age and sex. For this study, we were only able to acquire female ferrets that were sexually intact at 1 year of age as well as sexually intact male ferrets at 1 year and 2 years of age. Then able to make two distinct comparisons, on where 1-year old females were compared to 1-year-old males and the other where 1-year-old males were compared to 2-year-old males. As ferrets reach sexual maturity by 8-months-old, 1-year-old ferrets correspond to adults^53^. By age 6 ferrets are considered elderly, making a 2-year-old ferret correspond to middle age in humans^54^.

**Figure 1:**
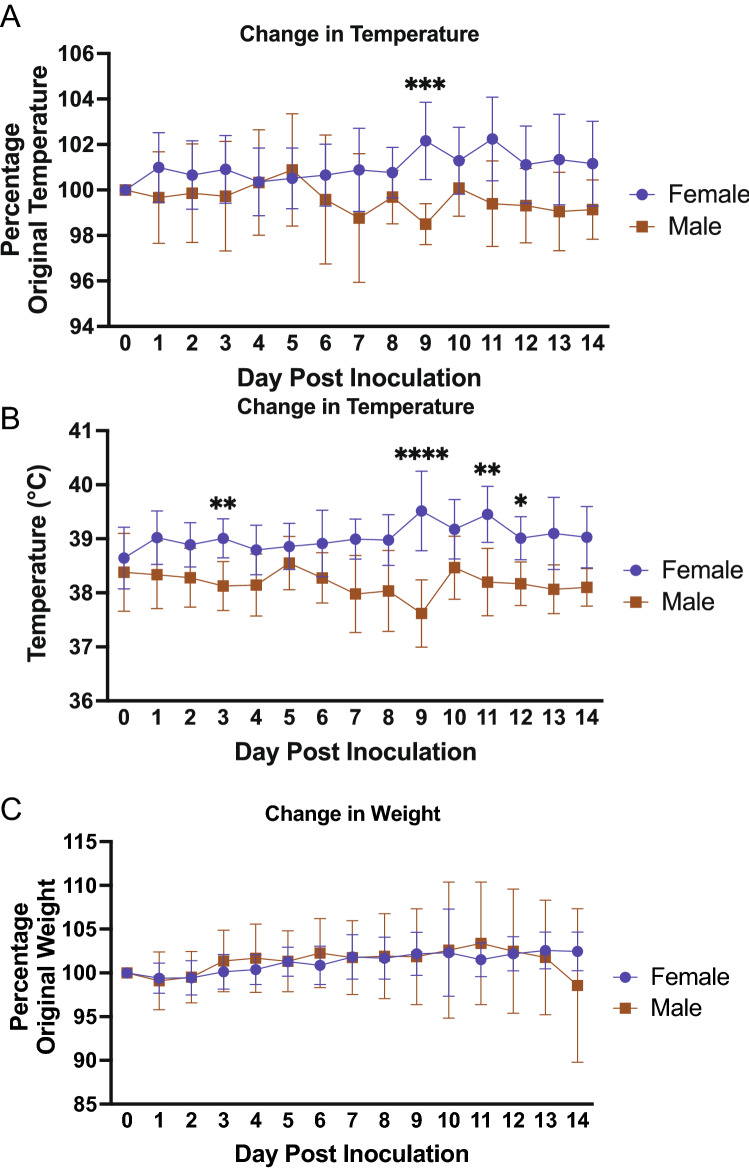
Intranasal SARS-CoV-2 infection in sexually intact ferrets leads to significantly lower temperature in males compared to females. (**A**) Sexually intact 1-year-old female and 1-year-old male ferrets were intranasally inoculated with the Severe Acute Respiratory Syndrome coronavirus 2 (SARS-CoV-2) (10^6^ TCID_50_) and weight was recorded for 14 days post inoculation and represented as percentage of original temperature and (**B**) raw temperature. (**C**) Weight was also measured for 14 days after inoculation and represented percentage of original weight. Results show the mean of at least 6 ferrets per group. Results show the mean of at least 6 ferrets per group. *A p-value less than 0.05 determined by ANOVA comparing females to males. Error bars indicate ± standard deviation (SD).

**Figure 2:**
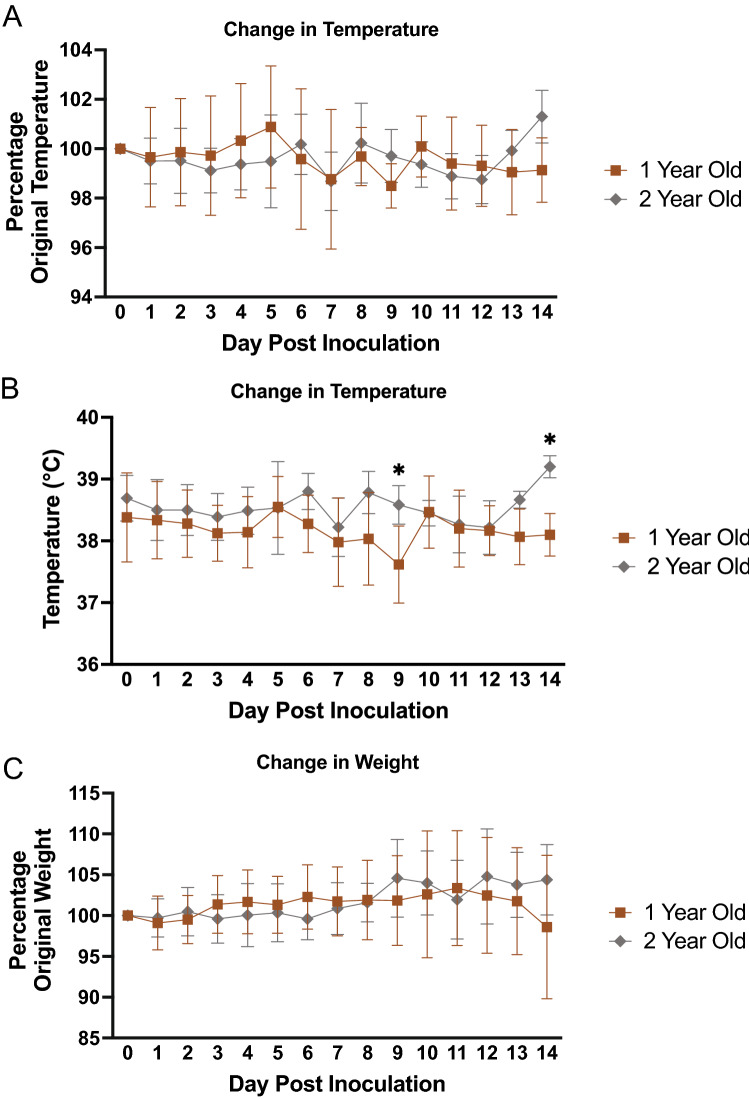
Intranasal SARS-CoV-2 infection in ferrets leads to significantly higher temperature in 2-year-old animals compared to 1-year-old animals. (**A**) 1-year-old and 2-year-old male ferrets were intranasally inoculated with SARS-CoV-2 (10^6^ TCID_50_) and temperature was also analysed for 14 days after inoculation and represented as a percentage of original temperature and (**B**) raw temperature. (**C**) Weight was recorded for 14 days post inoculation and represented as percentage of original weight. Results show the mean of at least 6 ferrets per group. *A p-value less than 0.05 determined by ANOVA comparing 1-year-old animals to 2-year-old animals. Error bars indicate ± SD.

All three groups were intranasally inoculated with 10^6^ TCID_50_ of SARS-CoV-2 while anesthetized. Clinical responses such as weight loss, temperature, activity, and other signs of neurological and gastrointestinal disease were monitored for 14–21 days post inoculation (pi). In-life nasal washes, blood samples and tissues were collected on days 2, 5, 7, 14, and 21 pi to analyse host responses and viral burden among the groups. Ferrets display human-like clinical symptoms after viral infection^55^. Weight and temperature were monitored after inoculation for 14 days in Figs. 1 and 2, for the sex and age comparison, respectively.

Significant variability was observed for temperature between the 1-year-old male and 1-year-old female comparison (Fig. 1). Female ferrets experienced temperature increases after inoculation compared to their baseline values (Fig. 1A). On day 1 pi, female temperature increased to 101% of original temperature that was sustained until day 4. There was again a noticable increase in the female temperature on day 9 when temperature was recorded at 102% of original readings. Although this temperature increase was mild in the females, it differed significantly from the 1-year-old male ferret groups. The male ferrets experienced a temperature decrease after inoculation (98–99% of original temperature). The temperature recordings in degrees Celsius are shown for comparison and supported the trend of decreased temperature in the 1-year-old male ferrets compared to the females which had an increase (Fig. 1B). Minimal clinical changes in weight were observed for all groups throughout the time course. Both groups remained close to their original weight (100%) and no statistical differences were noted (Fig. 1C). We were able to observe uninfected ferrets over a course of 7 days to gain a large baseline range for percent temperature change (Fig. 1A), degrees temperature fluctuation (Fig. 1B), and percentage of weight change (Fig. S1C). Additionally, we have previously published temperature and weight ranges for uninfected ferrets^56^. When we directly compared the 2-year-old male ferrets (represented by the grey line on the graph) to the 1-year-old male ferrets (represented by the brown line), similar temperature readings were found between the two groups with minimal days at statistically different temperatures (Fig. 2A,B). Considering control uninfected values, the temperature and weight change were within normal ranges for ferrets. However, we did find a consistent temperature trend for the 1-year-old male and 1-year-old female, where the males were consistently and statistically lower in temperature compared to the females.

### Increased durability of viral load in 1-year-old males vs 1-year-old females and in 2-year-old males compared to 1-year-old males

**Figure 3:**
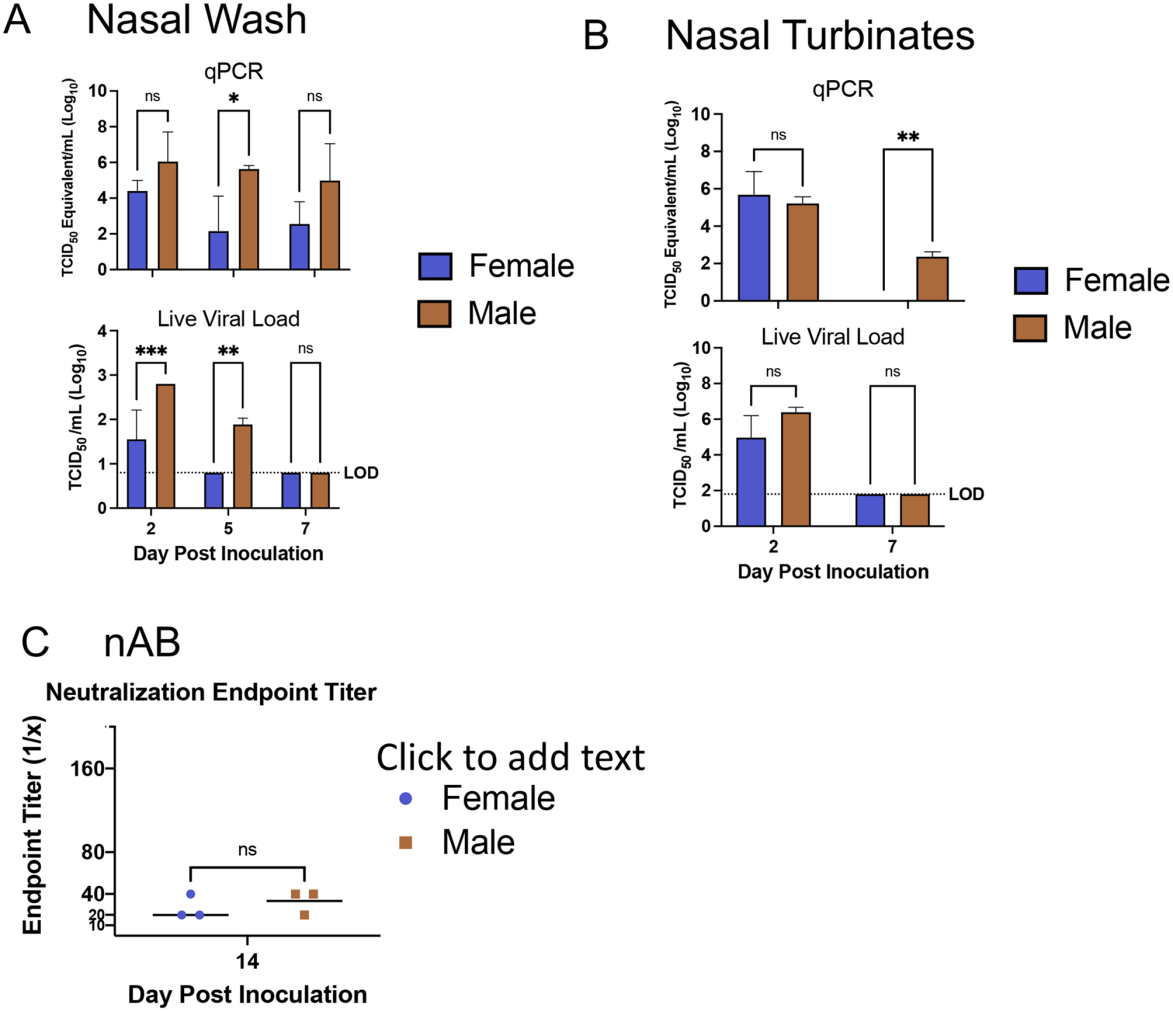
Increased SARS-CoV-2 viral presence occurs after infection in upper respiratory tract of male ferrets. (**A**) Nasal washes were collected from male and female ferrets to quantify SARS-Co-V-2 by qPCR and live viral load assay. Infectious titer of TCID_50_/mL was calculated using the Reed and Muench method. (**B**) vRNA and live virus was also quantified in the nasal turbinates. (**C**) Virus neutralization titers were determined by standard neutralization assays using plasma collected from both groups on day 14. LOD = limit of detection. Error bars represent SD. *A p-value of less than 0.05 as determined by ANOVA or Student’s t-test as appropriate. N is 3 for all groups.

**Figure 4:**
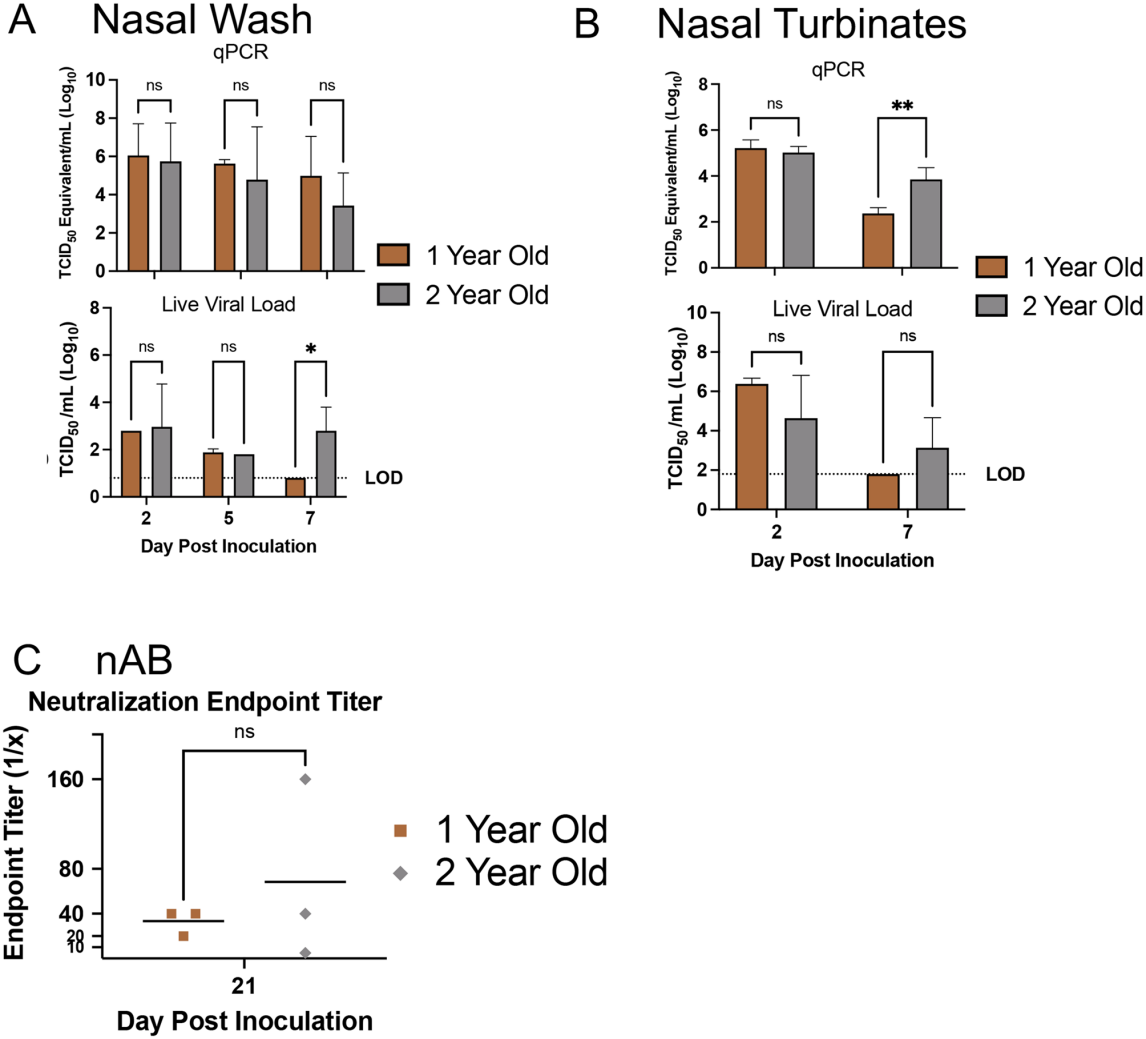
Prolonged SARS-CoV-2 viral presence occurs after infection in upper respiratory tract of 2-year-old ferrets compared to 1-year-old ferrets. (**A**) Nasal washes were collected from 1-year-old and 2-year-old ferrets to quantify SARS-Co-V-2 by qPCR and live viral load assay. Infectious titer of TCID_50_/mL was calculated using the Reed and Muench method. (**B**) vRNA and live virus was also quantified in the nasal turbinates. (**C**) Virus neutralization titers were determined by standard neutralization assays using plasma collected from both groups on day 21. LOD = limit of detection. Error bars represent SD. *A p-value of less than 0.05 as determined by ANOVA or Student’s t-test as appropriate. N is 3 for all groups.

It has been shown that males and seniors have increased SARS-CoV-2 viral burden compared to younger females^57,58^. We assessed the viral burden by qRT-PCR and TCID50 assays in the upper and lower respiratory tracts of our ferret groups at virologically important time points following SARS-CoV-2 infectionby age-matched and sex-matched comparisons, (Figs. 3 and 4, respectively). Assessment of nasal washes was used to determine the temporal dynamics of viral load and shedding. Infectious virus was present in the nasal washes of 1-year-old female ferrets on day 2 pi though vRNA determined by qRT-PCR persisted until day 7 pi (Fig. 3A). The vRNA values in the nasal washes from females was ~ 4 TCID_50_ Equivalent/mL (Log10) on day 2 but decreased to below 2 TCID_50_ Equivalent/mL (Log10) which was significantly lower compared to those of 1-year-old males at ~ 5 TCID_50_ Equivalent/mL (Log10) at day 7 pi. Live viral load was significantly greater in the 1-year-old males on both day 2 and day 5 pi. Similar trends were found in the nasal turbinate tissue where 1-year-old male ferrets had statistically higher vRNA levels on day 7 pi and female tissue was negative for vRNA (Fig. 3B). No stastical differences were noted for neutralizing antibody (nAb) titer between in 1-year-old males and 1-year-old females at day 14 pi (Fig. 3C). From this data, the natural history of the viral load in the upper respiratory tract can be suggested to reach apex for female 1-year-old ferrets on day 2 pi and have resolved by day 5 pi where as for 1-year-old males resolution occurs by day 7 pi.

Conversely, when comparing 1-year-old and 2-year-old males, infectious virus was shed in nasal washes until day 7 for the older male group where as no infectious virus was detected on day 7 for the 1-year-old males which was statistically significant (Fig. 4A). Within the nasal turbinate tissue, increased vRNA was detected in the older male group on day 7 along with detectable infectious virus which was not found in the 1-year-old males at this time (Fig. 4B). All animals had detectable virus neutralizing titers except for one in the 2-year-old male group (Fig. 4C). In regard to the viral load natural evolution in the upper respiratory, 2-year-old male ferrets reach viral apex on day 2 pi but resolve the viral infection after day 7 pi.

We assessed vRNA and infectious virus presence in tissues outside of the upper respiratory tract including salivary glands, trachea, lung lobes (right cranial, right middle, right caudal, left cranial, left caudal, and accessory lobes), mediastinal lymph nodes, heart, kidney, liver, spleen, and large intestine (colon) for the 1-year-old males versus 1-year-old females and the 1-year-old males versus 2-year-old males (Fig. S2A,B, respectively) to determine if virus tropism was expanded outside the upper respiratory tract. Viral RNA was noted in the lung lobes and trachea as well as in the large intestine for all groups at some point in the sampling days. No statistical differences were found in respect to vRNA quantity between experimental groups. No infectious virus was cultured from any vRNA positive tissues (data not shown). Together, this data suggested that infectious viruses was mainly contained to the upper respiratory tract.

**Figure 5:**
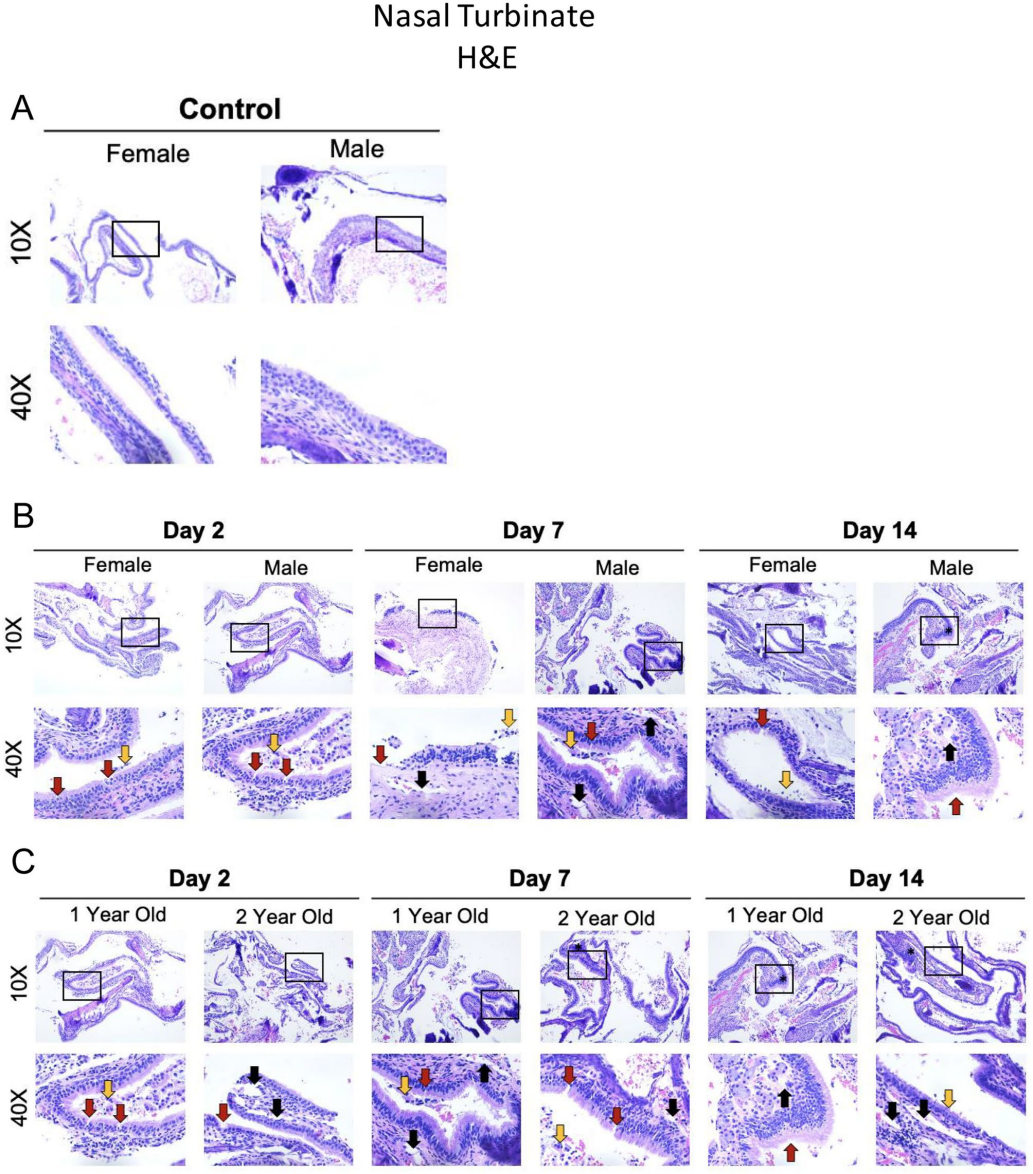
2-year-old male ferrets inoculated with SARS-CoV-2 have increased damage in the nasal turbinates compared to 1-year-old ferrets. (**A**) Nasal turbinates were examined by H&E collected from uninfected control ferrets. (**B**) Histopathology of H&E-stained nasal turbinates were also analyzed in infected female (1-year-old) versus male (1-year-old) ferrets and (**C**) 1-year-old versus 2-year-old ferrets. The collected nasal turbinates were formalin-fixed and paraffin block embedded prior to slide mounting and staining by H&E. Stained tissues were visualized and images captured via the Leica DMI100 Brightfield microscope and camera. Images were captured at 10× and 40×. Images shown are representative of three animals per group, per timepoint. Black arrows indicate cavity formation; red arrows denote epithelial erosion; and orange arrows point to epithelial cell sloughing. Lines refer to loss of ciliated cells.

Due to the differences in the upper respiratory tract we observed across the ferret groups in both vRNA and infectious virus, we assessed infection-mediated pathology within nasal turbinate tissues. Uninfected male and female tissue is shown in Fig. 5A. The infected 1-year-old males versus infected 1-year-old females (Fig. 5B) and infected 1-year-old males versus infected 2-year-old males (Fig. 5C). Turbinates from 1-year-old female ferrets had minimal immune cell infiltration and tissue architecture destruction day 2 pi (three of three animals) (Fig. 5B). Ciliated epithelial cells were visible at this time point with minimal cilia loss (three of three animals). Mononuclear cell infiltration was first detected on day 7 (two of three animals) and was most pronounced on day 14 pi (three of three animals) as indicated by the asterisk in the females. Rhinitis, inflammatory cell infiltration, and loss of ciliated cell layers are evident at this time (three of three animals). Granulocytes can also be seen from days 7 to 14 pi (three of three animals). One-year-old males had increased ciliated epithelial cell loss starting on day 2 pi (two of three animamls) (indicated by red arrows) compared to females (Fig. 5B). Cavity formation was first noted in this male group on day 7 (three of three animals) (black arow) with concomitant edema (three of three animals) and presence of immune exudate (three of three animals), monocytic cell infiltrate (three of three animals), disruption of cilia (two of three animals), and cavitation (three of three animals), and heavy sloughing (three of three animals). Significant mononuclear cell infiltration (three of three animals), as well as signs of necrosis (three of three animals), was evident in 1-year-old males by day 14 pi as indicated by the asterisk (Fig. 5B).

Tissue pathology of the 1-year-old males was then compared to the nasal turbinates from 2-year-olds. This older male group more significant epithelial destruction and immune cell infiltration compared to the 1-year-old males (Fig. 5C). Evidence of mononuclear cell infiltration was identified as early as day 2 pi (three of three animals) (asterisk) and remained visible throughout the time course (three of three animals). Significant loss of ciliated cells was observed on day 2 pi (black line) (three of three animals) with signs of erosion in the surface of the epithelial cells (red arrow) (three of three animals), as well as cavity formation (black arrow) (three of three animals), necrosis (three of three animals), and immune infiltration (three of three animals). Erosion could be seen on days 2 and 7 pi whereas cavity formation in the submucosa was evident on days 7 and 14. Exocytosis of mononuclear cells was consistently observed post infection (three of three animals). Orange arrows point to epithelial cell sloughing.

**Figure 6:**
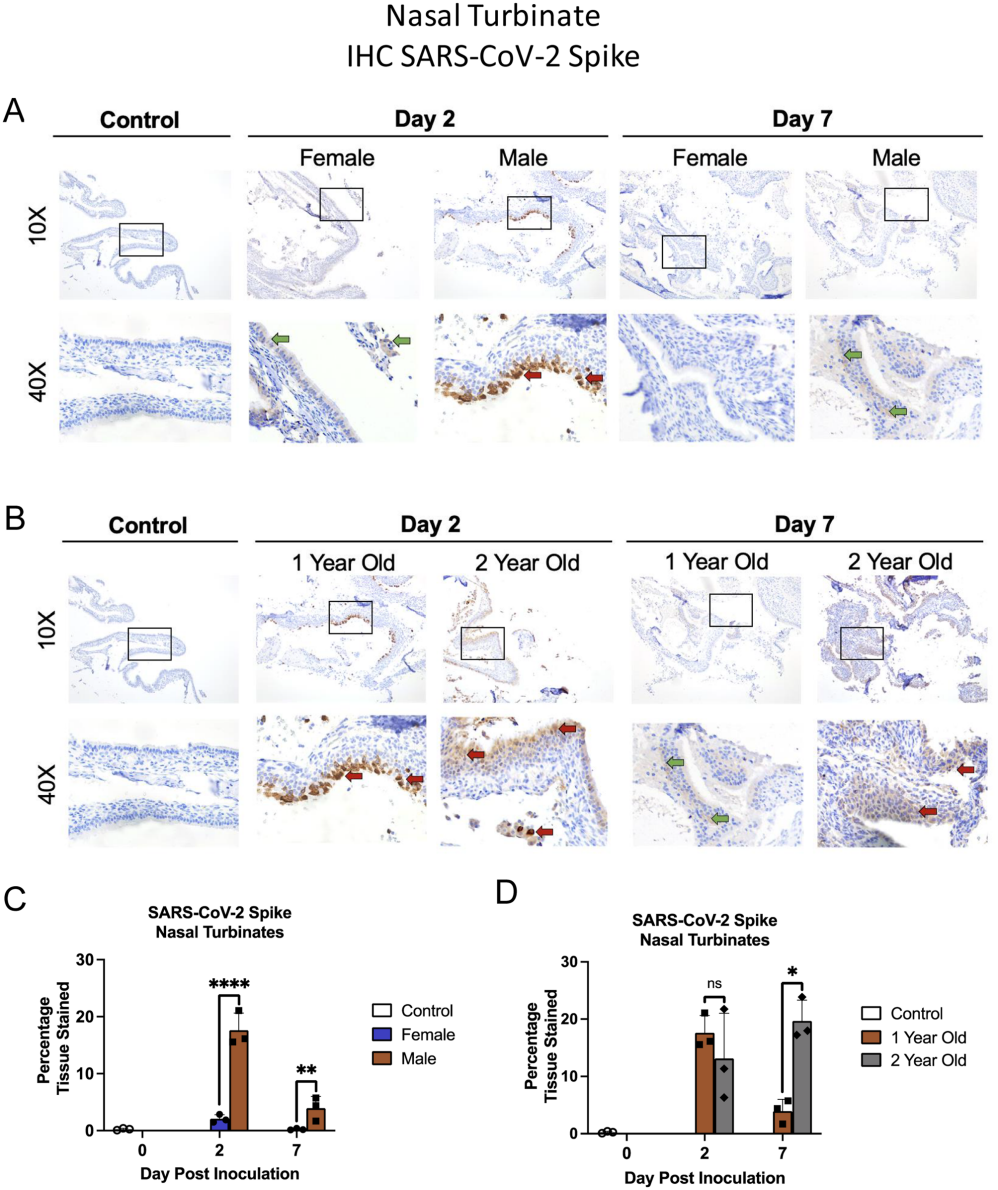
2-year-old male ferrets have increased and prolonged SARS-CoV-2 viral antigen presence in nasal turbinates. (**A**) Formalin-fixed nasal turbinates collected from uninfected, control ferrets as well as female and male ferrets on days 2 and 7 post SARS-CoV-2 inoculation, were stained for SARS-CoV-2 spike protein. (**B**) Formalin-fixed nasal turbinates collected from uninfected, control ferrets as well as 1-year-old and 2-year-old ferrets on days 2 and 7 post SARS-CoV-2 inoculation, were stained for SARS-CoV-2 spike protein. Red arrows indicate high amounts of viral antigen staining and green arrows indicate low amount of viral antigen staining by immunohistochemistry (IHC) using an anti-spike rabbit monoclonal antibody. Stained tissues were visualized, and images captured using a Leica DMI100 Brightfield microscope and camera. Images were captured at 10× and 40×. Images shown are representative of three animals per group, per timepoint. (**C**) Staining was quantified utilizing Image-J in age matched female and male ferret nasal turbinates (**D**) and 1-year-old and 2-year-old ferret using scans of entire tissue captured by Aperio ScanScope XT slide scanner. Error bars represent SD. *A p-value of less than 0.05 as determined by ANOVA. Three animals were analyzed at each time point for all groups.

We next investigated viral antigen presence and location within the tissue by IHC for 1-year-old males versus 1-year-old females (Fig. 6A) and 1-year-old males versus 2-year-old males (Fig. 6B). Viral antigen staining was then quantified (Fig. 6C,D). 1-year-old males had a high concentration of positively stained cells in the outer layer of the nasal olfactory epithelium on day 2 (three of three animals), (indicated by red arrows) which diminished by day 7 (green arrows). Comparatively, 1-year-old females had minimal viral antigen staining in the pseudostratified columnar epithelium which was only observed on day 2 pi (three of three animals) (Fig. 6A, indicated by green arrows). When the viral antigen was quantified and compared for the two groups, 1-year-old females had statistically less percentage of tissue stained compared to 1-year-old males (Fig. 6C). Two-year-old males were next compared to the 1-year-old males and similar levels of viral antigen were noted on day 2 pi in the two groups although the distribution and intensity differed (Fig. 6B). Intense staining was found in the outer epithelial layer in the 1-year-old males where has the staining was more diffuse for the 2-year-old males but extended to deeper layers of the epithelium (red arrows). Staining was still prominent in the older group on day 7 pi which was found to be ~ 15% greater than the 1-year-old males after quantification (Fig. 6D).

Together, all infected ferret groups showed damage to the nasal turbinates over the course of infection with evidence of viral antigen within the tissue. One-year-old males and 2-year-olds had early signs of mononuclear cell infiltration and cavity formation with damage to the tissue throughout the time course. Tissue analysis of viral antigen revealed more extensive, and a longer duration of, antigen in the 2-year-olds which was consistent with our viral load findings.

### Interferon stimulated gene upregulation in the nasal turbinates was delayed in 2-year-old ferrets

**Figure 7:**
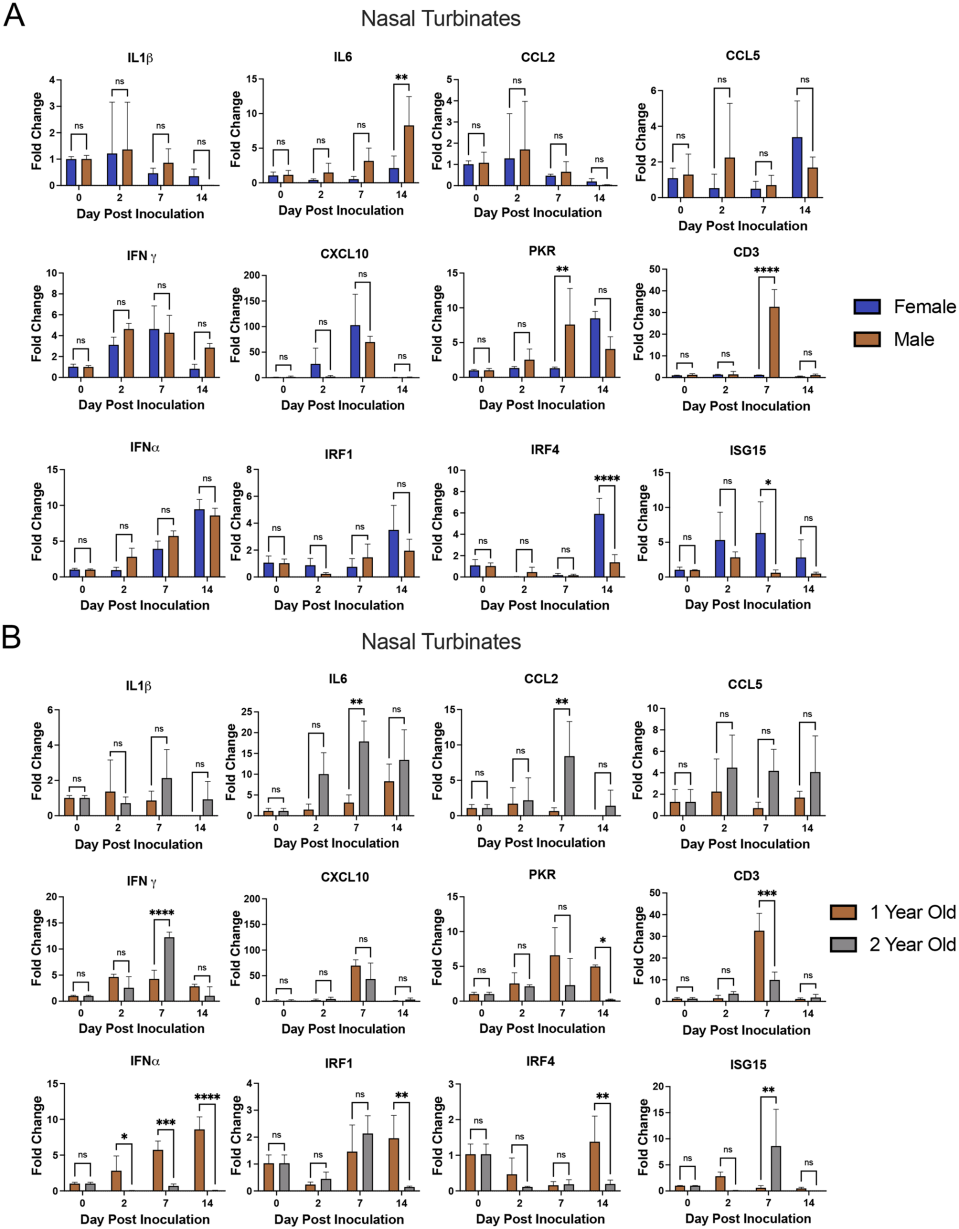
RNA expression analysis by qRT-PCR indicated increased IL-6 but decreased IFN response in the nasal turbinates of 2-year-old male ferrets post SARS-CoV-2 infection. (**A**) qRT-PCR analyzing the expression of specific immune response genes was performed on RNA extracted from nasal turbinate of age matched female (1-year-old) and male (1-year-old) ferrets. (**B**) qRT-PCR was again performed on RNA extracted from nasal turbinate of sex-matched male ferrets at 1-year-old and 2-years-old. In both analyses, samples were assessed for IL-1 beta, IL-6, CXCL10, CCL2, CCL5, IFN-gamma, CXCL10, PKR, CD3, IFN-alpha, IRF1, IRF4, and ISG15 with specific ferret primers (Table 1). Fold change was calculated via ΔΔCt against controls with BACT as the housekeeping gene. Error bars represent SD. Three ferrets per group were used for the analysis of all time points. *A significant difference as determined by Student’s t-test comparing against female as control.

To acquire insight into the mechanisms related to viral load increases in the male ferrets which was further increased in the 2-year-old ferrets, we performed qRT-PCR using a panel of primers we have previously validated in ferrets^23,24,43^. The expression of antiviral genes, inflammatory genes, and immune cell markers were investigated and the results analyzed by sex (age-matched) (Fig. 7A) and by age (sex-matched) (Fig. 7B). In the age-matched analysis, no differences were noted for the antiviral genes (IFN-gamma; IFN-alpha; CXCL10; or IRF-1) whereas the inflammatory marker IL-6 and CD3 were statistically increased in the males. Notable increased expression of IRF4 and ISG15 were seen in the 1-year-old females. Interestingly when this immune panel was applied to the sex-matched comparisons, the 2-year-old males had statistical increases in the inflammatory genes IL-6 and CCL2 compared to the younger males and the younger males had significant regulation of type 1 interferon responses (IFN-alpha, IRF-1, and IRF-4) over the 2-year-old males. As representative markers of the inflammatory response and interferon response, IL-6 and CXCL10, respectively, were examined in the lung tissue per group comparison (Fig. S3A,B). Similar trends were observed for these markers in the lungs that were seen in the nasal turbinates.

**Figure 8:**
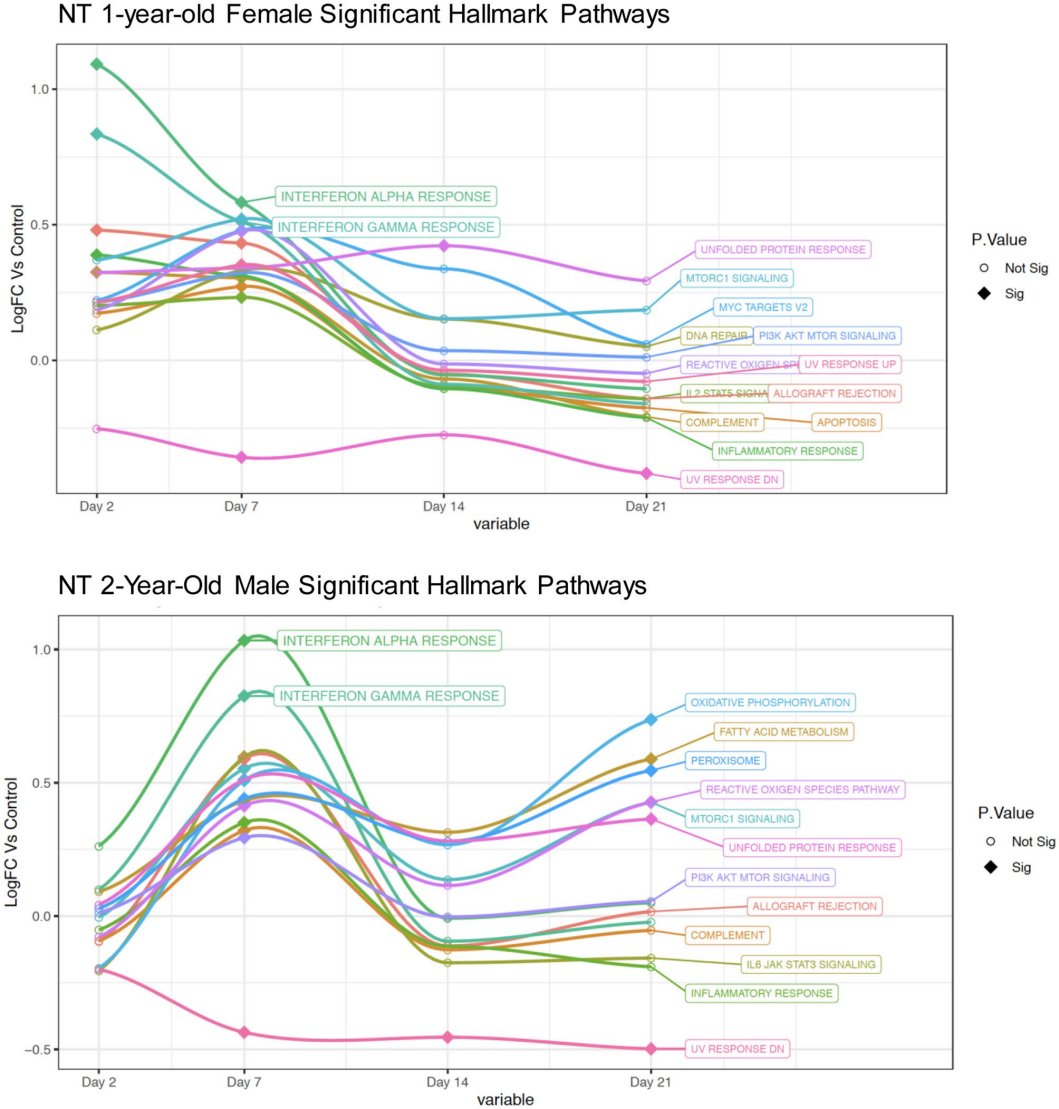
Unique signaling pathway regulation for 2-year-old male ferrets and 1-year-old female ferrets in the upper respiratory tract after SARS-CoV-2 infection. The RNA extracted from the nasal turbinates of 1-year-old females and 2-year-old males at time points post infection with SARS-CoV-2 was sequenced using the Illumina platform. Longitudinal enrichment analysis was independently performed at plotted per experimental group for days 0, 2, 7, 14, and 21 pi. (**A**) Enrichment profiles were created using Hallmark Pathways over the time course for 1-year-ol female ferrets. (**B**) The Hallmark enrichment profiles were plotted over the time course for 2-year-old ferrets. Signaling pathways which were statistically changed (FDR < 0.10) compared to baseline for at least one time point were included: IFN-alpha, IFN-gamma, Unfolded Protein Response, MTORC, MYC Targets V2, DNA Repair, PI3K AKT MTOR Signaling, Reactive Oxygen Species, UV Response, IL2 STAT5 Signaling, Allograft Rejection, Apoptosis, Inflammatory Response, UV Response DN, Peroxisome, Oxidative Phosphorylation, IL6 JAK STAT3 Signaling, and Complement. Filled diamonds represent a time point statistically changed compared to baseline. Open circles represent time points that are not statistically significant compared to baseline. Three ferrets per group per time point were analyzed with the exception of a poor sample in the female on day 7 which did not pass quality control.

We next performed RNAseq analysis for only the 1-year-old females and 2-year-old males due to experimental constrainst experienced during the initial phase of the pandemic. We performed unbiassed, whole transcriptome transcriptome analysis. RNA was isolated from upper (nasal turbinates) and lower (right cranial and caudal lung lobes) respiratory tracts of control animals, infected female ferrets and 2-year-old ferrets at days 2, 7, 14, and 21 following inoculation. Gene enrichment analysis plots analyzing each group independently, leveraging the Hallmark Signalling Pathway collection^59,60^ indicated different longitudinal profiles of several prominent signalling pathways for the SARS-CoV-2 infected 1-year-old females and 2-year-old males (Fig. 8). Most noted were the early increase in interferon-alpha (IFN-α) and interferon-gamma (IFN-γ) for the 1-year-old females on day 2 pi (Fig. 8A) whereas the 2-year-old males had no expression of interferons on day 2 which peaked later on day 7 pi (Fig. 8B). Also of note were the decreasing trends of inflammatory response associated pathways in the 1-year-old females on day 21 of reactive oxygen species, IL2 STAT5 signalling, complement, inflammatory responses, allograft rejection, and apoptosis (< 0.1). Conversely, 2-year-old males had statistical increases in pathways involved with inflammatory responses including oxidative phosphorylation, fatty acid metabolism, peroxisome, reactive oxygen species, MTORC and unfolded protein response (< 0.1 and < 0.05 (data not shown)). Once we had gained a perspective of the overall pathway level changes over time, we looked more closely at the top 50 DEGs by p-value (all p < 0.05) in isolation by day post-infection to identify the individual genes represented in each pathway (Figs. S4, S5 and S6). Interferon response and antiviral response genes most highly expressed in the female day 2 nasal turbinates included OASL, MX1, DHX58, RSAD2, ISG15, and CTSG. By day 7 pi, 2-year-old ferrets had a shift in gene profiles with significant increases in CXCL9, CXCL10, CXCL11, OASL, RSAD2, IRF7, ISG15, ISG20, CAMP, and CTSG (Fig. S4). Similar trends were also seen in analysis of the right cranial (Fig. S5) and right caudal (Fig. S6) lung lobes.

## Discussion

Here we investigated the influence of age and sex on clinical outcomes and host responses after SARS-CoV-2 infection using the ferret model. Importantly, by using ferrets with intact sex organs, we were able to properly evaluate the variable of sex and its influence on infection and host responses. Sex as well as aged appeared to have significantly affected viral load in the upper respiratory when we compared age-matched males and females and then sex-matched 1-year-old males to 2-year-old males. Host gene expression analysis suggested that Inflammation was prolonged in the males compared to females whereas increased age decreased the interferon response in the 2-year-old males compared to younger males. Further, RNAseq analysis showed delayed expression of antiviral genes such as MX1, IRF7, and ISG15 in the 2-year-old males. COVID-19 caused by SARS-CoV-2 has significantly impacted both the male sex and older individuals. Our findings may inform human studies and suggest investigation of the interrelationship of viral shedding and interferon signalling responses in COVID-19 patients which may be biased in males and the older age groups. The identification of prolonged viral shedding in older males may have implications in public health responses and the analysis of transmission events.

To study the contribution of sex and age to the development of severe COVID-19, we were able to parse the variables of age and sex to stratify clinical, virological, and immune outcomes post SARS-CoV-2 infection by utilizing ferrets with intact sex organs. Since, previously reported ferret SARS-CoV-2 studies using younger fixed ferrets indicated a mild disease, we hypothesized that older sexually-intact male ferrets would develop severe disease post SARS-CoV-2 infection more similar to humans^32,33,48–50,61^. Since older males are at higher risk of hospitalization and death from SARS-CoV-2 infection, we anticipated the establishment of a preclinical model with a severe COVID-19 phenotype that could be employed for vaccine and therapeutic development. In contrast, we found that none of our older or sex organ intact ferrets developed significant weight loss after SARS-CoV-2 infection, but temperature was significantly different between age-matched males and females. Given that the normal temperature range for ferrets is 37.7–39.4 °C^62^, we found that females developed a sustained mild fever similar to that reported for ferrets infected with SARS-CoV-2 in other studies^63^. Conversely, all males in our study experienced minimal decreases in temperature. Although, temperature has been shown to be dysregulated in older patients infected with other respiratory viruses, we found temperature responses to be more associated with the sex of the ferret. Since our ferrets were older but not yet considered to be senior ferrets, studying older ferrets may have led to different responses. Our results suggest older male COVID-19 patients may fail to be identified in early stages because of atypical clinical pictures. Currently, there are limited clinical definitions of COVID-19 per age group and sex. Defining the clinical picture for specific cohorts will be essential for proper diagnosis, treatment plans, and patient outcome.

Although SARS-CoV-2 continues to circulate globally, little is understood regarding host-specific viral dynamics and transmission. In our preclinical study, we found that there was a sex and age influence on SARS-CoV-2 in the upper respiratory tract and not the lower respiratory tract possibly due to distribution of the host receptors ACE2 (angiotensin-converting enzyme 2) and TMPRSS2 (a serine protease) in ferrets^64,65^. Previous studies have indicated that ferrets do express ACE2 in their lower respiratory tract^66^ but the expression of the second host factor that is sometimes required for virus entry has not been investigated in ferrets. Male ferrets shed live SARS-CoV-2 virus longer than females and shedding virus from males increased by age. Although there were statistical differences in the shedding of infectious virus on day 2 pi, analysis of vRNA and live virus in the nasal turbinate tissue on this day did not indicate a quantitative difference of viral burden by sex or age, indicating that females and males were equally susceptible to infection and viral replication. Therefore, the differences that were observed among the groups in respect to virus durability and prolonged shedding conversely suggested there was a sex and age bias in the ability to control viral infection over time. We did find that the 1-year-old female ferrets were only positive for infectious virus in either nasal wash or nasal tissue on day 2 pi where the males continued to be positive on day 5 (infectious virus and vRNA) and day 7 (vRNA) suggesting that the presence of female sex hormones or sex organs in our sexually intact female ferrets influenced the efficiency of viral clearance. Furthermore, the viral loads we detected in 2-year-old ferrets were similar to those reported by other SARS-CoV-2 ferret studies (viral clearance ~ day 8 pi)^33^. If the ferrets in this study were neutered or spayed as it typical for research ferrets, our data suggests that younger age and the presence of sex organs may be beneficial to efficiently clearing virus. In respect to human COVID-19, our results seemed to recapitulate human SARS-CoV-2 viral load demographics where older people as well as males have been tested to be SARS-CoV-2 positive for longer periods of time^57,58^. Mouse models of SARS-CoV-1 as well as SARS-CoV-2 also indicated that viral burden in the respiratory tract of males and older animals is significantly increased^39,67,68^ suggesting similar influence of age and sex for SARS-like coronaviruses. In contrast to these findings, a preclinical study investigating the effect of age on SARS-CoV-2 morbidity using the Syrian hamster model found no difference in viral shedding of older and younger animals^69^. This report may indicate that although the Syrian hamster model recapitulates lung disease associated with human COVID-19^37^, ferrets and mice may be a superior model for age-based studies.

We found an association between altered immune responses with prolonged viral load in 2-year-old ferrets characterized by a significant delay in interferons and interferon stimulated genes. These gene sets were robustly induced in the 1-year-old female ferrets at our early time point of day 2 pi. In another study in ferrets infected with SARS-CoV-2, the host response was investigated in the nasal washes and trachea in 4 month old castrated male ferrets^70^. In this study, the ferrets were de-sexed and also considerably younger than the ferrets used for our work. Lower magnitudes of gene regulation was noted in this study after infection compared to ferrets infected with the H1N1 virus A/California/07/2009 although immune response genes such as CCL2, CCL8, CXCL9, and CXCR1 increased over the time course. We also found lower levels of immune response gene regulation than what we would have expected. The human COVID-19 demographics clearly indicate that older males are at higher risk of severe COVID-19 but this data does not give insight into the factors influencing disease. In respect to sex biases, biological and behavioural factors may both influence disease demographics^71^. Our results leveraging a controlled preclinical animal model that removed behavioural factors suggested that biological sex strongly affected the outcome of SARS-CoV-2 infection. Recently, humoral responses as well as interferon signalling have been implicated in the sex biases of viral infection in humans. Data from severe COVID-19 patients indicated that males and the elderly had increased neutralizing antibodies toward SARS-CoV-2, possibly suggesting a pathogenic humoral involvement or mechanism affecting antibody production during infection^50^. Several other human and animal studies have reported that perturbed interferon responses are associated with SARS-CoV-2 infection, suggesting that dysregulated interferon signalling is a common mechanism driving disease^61,70,72^. A report by Bastard and colleagues showed a significant number of autoantibodies elicited toward type I interferons in humans may lead to interferon dysregulation as a possible mechanism^61^. Previous studies have also suggested a role for Toll-like Receptor 7 (TLR7) in sex-biased interferon signalling during viral infections^61^. TLR7, a viral nucleic acid pathogen recognition receptor, is located on a region of the X chromosome which is known to have a lower level of chromosomal inactivation, allowing females to have stronger interferon responses during viral infection due to gene dosing^49,73–75^. Taken together, these reports may represent mechanisms contributing to the prolonged viral presence and subsequent dysregulated interferon responses we found in the upper respiratory tract of older males. Future studies should explore the sex-specific regulation of interferon responses with regard to the involvement of type I interferon-specific antibodies or TLR7 signalling in viral replication and clinical morbidity^61,74,76^.

Our study was limited by lack of an older female ferret group, due to lack of a supply of these animals early in the pandemic. Therefore, our conclusions focus on the suggestion that early antiviral intervention may be more beneficial for males compared to females infected with SARS-CoV-2. Moreover, 2-year-old male ferrets had higher viral load that persisted longer in their upper respiratory tracts compared to 1-year-old males and that in turn was associated with uniquely delayed antiviral gene expression. While further research is needed to confirm the target genes we have identified, our study represents a unique transcriptomic resource and established animal model for sex as a biological variable for further research. Additional sex-aggregated analysis of antiviral use in clinical studies may be beneficial to understanding the outcome of antiviral treatment during COVID-19 for compounds such as remdesivir or interferon-beta. More work is needed to expand on these conclusions and identify whether these mechanisms are also at play in humans and if older males may be a cause more transmission due to prolonged viral shedding. Our model which shows sex- and age-specific regulation of the interferon responses during SARS-CoV-2 could be used for the investigation of sex-specific effectiveness of interferon-beta or interferon-lambda treatment.

## Methods

### Ethics statement

All work was conducted in accordance with the Canadian Council of Animal Care (CCAC) guidelines. The study was also approved by the University Animal Care Committee (UACC) Animal Research Ethics Board (AREB) from the University of Saskatchewan in association with the Vaccine and Infectious Disease Organization (VIDO) under the AUP number 20200016. For ferret manipulation, 5% isoflurane anesthesia was used with all efforts to minimize suffering. All work was carried out in compliance with the ARRIVE guidelines.

### Virus

The SARS-CoV-2 isolate/Canada/ON/VIDO-01-2020 used for infections and in vitro assays was isolated from a patient presenting at a Toronto hospital upon returning from Wuhan, China^77^. The viral stock grown in vDMEM (DMEM (Dulbecco’s Modified Eagle Medium) (*Wisent Bioproducts (Cat # 319-005-CL)*), 2% fetal calf serum (*Wisent Bioproducts (Cat # 090-150)*), 5 mL 100 × Penicillin (10,000 U/mL)/Streptomycin (10,000 μg/mL), and 2 μg/mL TPCK-trypsin) was from the second passage (GISAID-EPI_ISL_425177). All work with SARS-CoV-2 live virus was performed in a CL3 facility at VIDO-InterVac (Saskatoon, Saskatchewan, Canada).

### Animals, infections, and tissue collection

Female (1 year), adult male (1 year) and 2-year-old (2 years) ferrets with intact sex organs were purchased from Triple F Farms (Gillett, PA, USA). Ferrets were anesthetized for intranasal SARS-CoV-2 at 10^6^ TCID_50_ infections. On day 2, 7, 14, and 21 post infection, ferrets were terminally bled by intra-cardiac puncture and humanely euthanized. Three additional uninfected animals were monitored for weight and temperature fluctuations over 7 days, as controls. Following infection, weight, temperature, and clinical signs were monitored once daily for 14–21 days. Ferret weights were determined using a digital weight scale. Temperatures were measured by using a subcutaneous implantable temperature transponder (BioMedic Data Systems, Seaford, DE). Weight and temperature were calculated as a percentage of original weights.

### Viral titers

Nasal washes (1 mL) were collected post infection in anesthetized ferrets. Tissues collected at necropsy were homogenized in serum free DMEM using a Qiagen TissueLyzer. A 1:10 dilution series of sample was established in viral growth media (vDMEM) for TCID_50_ virus infection titration assays. Viral load was calculated by determining the 50% endpoint using the Reed-Muench method after observing cytopathic effect (CPE) on day 5 after cell inoculation.

### Viral neutralization assay

Plasma was heat-inactivated at 56 °C for 30 m and then serially diluted 1:2. Virus was diluted to 100 TCID_50_ in vDMEM and used at a 1:1 ratio to plasma, incubated at 37 °C for 1 h and added to cultured Vero-76 cells in 96 well plates for 1 h at 37 °C. Inhibition of CPE was observed and recorded on day 5 post infection.

### RNA extraction and quantitative real-time PCR (qRT-PCR)

Tissue RNA was extracted using the Qiagen RNeasy Mini kit cat. # 74106 (Qiagen, Toronto, Canada) according to the manufacturer’s instructions. vRNA was extracted from nasal washes using the Qiagen QIAamp Viral RNA Mini Kit cat. # 52904 (Qiagen). All host qRT-PCR was performed in triplicate on cDNA synthesized as previously described^43^ (Table 1). vRNA was quantified by Qiagen Quanti-fast RT probe master mix (Qiagen, Toronto, Canada) using primer/probe sets specific for the SARS-CoV-2 E gene. The reactions were performed on a StepOnePlus Real-Time PCR System in a 96-well plate (Thermo Fisher) as previously described^78^.

**Table 1:** qPCR primer sequences.

Target	Sequence		Source
BACT	F	TGACCGGATGCAGAAGGA	Paquette et al.^24^
	R	CCGATCCACACCGAGTACTT	
IL1β	F	GGACTGCAAATTCCAGGACATAA	Fang et al.^86^
	R	TTGGTTCACACTAGTTCCGTTGA	
IL6	F	AGTGGCTGAAACACGTAACAATTC	Fang et al.^86^
	R	ATGGCCCTCAGGCTGAACT	
CD3	F	GGCGGTGGCTGCAATC	Fang et al.^86^
	R	TCCAGTAATAGACCAGCAGAAGCA	
CCL2	F	GCCCAGCCAGATGCAATTA	Huang et al.^23^
	R	TTCTTTGGGACACTTGCTGC	
CCL5	F	GCTGCTTTGCCTACATTTCC	Fang et al.^86^
	R	CCCATTTCTTCTGTGGGTTG	
CXCL10	F	CCTCCTGTCCATGGTGTTCT	Huang et al.^23^
	R	TTTGGAATGAGGGTTCAAGC	
IFN α	F	TCTCCATGTGACAAACCAGAAGA	Paquette et al.^24^
	R	CAGAAAGTCCTGAGCACAATTCC	
IFNγ	F	TCAAAGTGATGAATGATCTCTCACC	Paquette et al.^24^
	R	GCCGGGAAACACACTGTGAC	
ISG15	F	AGCAGCAGATAGCCCTGAAA	Fang et al.^86^
	R	CAGTTCTTCACCACCAGCAG	
IRF1	F	CGATACAAAGCAGGGGAAAA	Fang et al.^86^
	R	GGCCTTGCACTTAGCATCTC	
IRF4	F	AATCCTCGTGAAGGAGCTGA	Fang et al.^86^
	R	AGATCCTGCTCTGGCACAGT	
PKR	F	ACGAATACGGCATGAAGACC	Fang et al.^86^
	R	TGGAAGGGTCAGGCATTAAG	

### Histopathology and immunohistochemistry

The tissues collected for histopathology were submersed in formalin for 1 week at 4 °C in CL3 and then incubated for an additional 24 h in formalin at in CL2 (4 °C) prior to histological processing. Formalin-fixed tissues were paraffin-embedded, sectioned, slide-mounted, and stained at Prairie Diagnostic Services (Saskatoon, Saskatchewan). For histopathology assessment, the tissue samples were stained with hematoxylin and eosin. To determine the localization of viral antigen within the respiratory tract, fixed and mounted tissue sections were analyzed by immunohistochemistry (IHC). Immunostaining to detect SARS-CoV-2 antigen was performed using VIDO-InterVac’s polyclonal rabbit anti-SARS-CoV-2 AS20-014. Immunohistochemical staining was conducted at Prairie Diagnostic Services, Saskatoon, SK, using an automated slide stainer (Autostainer Plus, Agilent Technologies Canada, Mississauga, ON). Antigen retrieval was performed in a Tris/EDTA pH 9 buffer at 97 °C for 20 min. The primary antibody AS20-014 was incubated for 30 min at a 1:400 dilution. Binding of the primary antibody was detected using an HRP-labelled polymer detection reagent (EnVision + System—HRP Labelled Polymer, Agilent Technologies Canada, Mississauga, ON). Images were captured using a Leica DMI100 Brightfield Microscope and DMC5400 20 MP color CMOS camera (Leica Microsystems, Concord, Ontario, Canada). To quantify IHC staining, scans of entire slides were taken using the Aperio ScanScope XT (Leica Biosystems, Nußloch, Germany). Images were processed in Image-J (U. S. National Institutes of Health, Bethesda, Maryland, USA). Each image was subject to ‘Colour Deconvolution’ as an ‘H DAB’ stain. Entire tissue area was measured utilizing ‘Colour 1’ at a threshold of 215, while stained portions were quantified utilizing ‘Colour 2’ at a threshold of 150. Staining threshold was chosen based on negative control slides. Staining was quantified as the percentage of entire tissue area where staining was detected, based on total tissue scans of 3 animals per time point and condition.

### RNAseq

RNA was assessed with a Fragment Analyzer (Agilent) using the Standard Sensitivity RNA kit. Total RNA was normalized to 100 ng prior to random hexamer priming and libraries generated using the TruSeq Stranded Total RNA—Globin kit (Illumina, San Diego, USA). Libraries were assessed on the Fragment Analyzer using the High Sense Large Fragment kit and quantified using a Qubit 3.0 fluorometer (Life Technologies). Sequencing was performed on a NovaSeq 6000 (Thermo Fisher) using a 50 base-pair, paired-end run for a minimum of 30 million reads per sample.

### RNAseq analysis

Raw demultiplexed fastq paired end read files were trimmed of adapters and filtered using the program skewer^79^ to remove any with an average phred quality score of less than 30 or a length of less than 36. Trimmed reads were aligned (HISAT2^80^) to the *Mustela putorius furo* NCBI reference genome assembly version MusPutFur1.0 and sorted using SAMtools. Reads were counted and assigned to gene meta-features using the program featureCounts^81^ (Subread package). Count files were imported into the R programming language and assessed for quality control, normalized, and analyzed using the limma-trend method^82^ for differential gene expression testing and the GSVA^83^ library for gene set variation analysis. Sequence and gene expression data are available at the Gene Expression Omnibus (accession number GSE160824). The GSVA algorithm was used to provide sample specific enrichment scores per pathway. The log2FC values represent the magnitude difference between the averages of the sample enrichment scores in each condition. The heatmaps were created by pheatmap (Pretty Heatmaps) and the line plots were created by ggplot2^84,85^.

### Statistical analysis

Unpaired, unequal variance, two-tail Student’s *t* test or one-way ANOVAs were conducted using GraphPad Prism8 (San Diego, USA). A *p* value of ≤ 0.05 was considered statistically significant.

## Supplementary Information

Supplementary Information.

Supplementary Figure S1.

Supplementary Figure S2.

Supplementary Figure S3.

Supplementary Figure S4.

Supplementary Figure S5.

Supplementary Figure S6.

## Data Availability

Sequence and gene expression data are available at the Gene Expression Omnibus (accession number GSE160824).
