# Supplementary material for: Sex and age bias viral burden and interferon responses during SARS-CoV-2 infection in ferrets
Source: Sci Rep. 2021 Jul 15;11:14536. doi: 10.1038/s41598-021-93855-9 (PMC8282673; doi:10.1038/s41598-021-93855-9)
Supplement: Supplementary file 1 — Supplementary Information. [file 41598_2021_93855_MOESM1_ESM.docx]

**Sex and age bias viral burden and interferon responses during SARS-CoV-2 infection in ferrets**

Magen E. Francis^1,2^, Brian Richardson^3^, Una Goncin^4^, Mara McNeil^1^, Melissa Rioux^1^, Mary K. Foley^1^, Anni Ge^1^, Roger D. Pechous^5^, Jason Kindrachuk^2,6^, Cheryl M. Cameron^7^, Christopher Richardson^1^, Jocelyne Lew^2^, Steven Machtaler^4^, Mark J. Cameron^3^, Volker Gerdts^2,8^, Darryl Falzarano^2,8^, and Alyson A. Kelvin^1,2,9,10,*^

**Affiliations**

^1^ Department of Microbiology and Immunology, Faculty of Medicine, Dalhousie University, Halifax, NS, B3H 4R2, Canada.

^2^ Vaccine and Infectious Disease Organization - International Vaccine Centre (VIDO-InterVac), University of Saskatchewan, Saskatoon, SK, S7N 5E3, Canada.

^3^ Department of Population and Quantitative Health Sciences, Case Western Reserve University, Cleveland, OH, 44106, USA.

^4^ Department of Medical Imaging, University of Saskatchewan, Saskatoon, SK, S7N 0W8, Canada.

^5^ University of Arkansas for Medical Sciences, Department of Microbiology and Immunology, Little Rock, AK, 72205, USA.

^6^ Laboratory of Emerging and Re-Emerging Viruses, Department of Medical Microbiology, University of Manitoba, Winnipeg, MB, R3E 0J9, Canada.

^7^ Department of Nutrition, Case Western Reserve University, Cleveland, OH, 44106, USA.

^8^ Department of Veterinary Microbiology, Western College of Veterinary Medicine, University of Saskatchewan, Saskatoon, SK, S7N 5B4, Canada.

^9^ Department of Pediatrics, Division of Infectious Disease, Faculty of Medicine, Dalhousie University, Halifax, NS, B3K 6R8, Canada.

^10^ Canadian Centre for Vaccinology, IWK Health Centre, Halifax, NS, B3K 6R8, Canada. [akelvin@dal.ca](mailto:akelvin@dal.ca).

***** Corresponding author:

Alyson A. Kelvin, PhD

Assistant Professor

Faculty of Medicine - Department of Pediatrics - Dalhousie University

IWK Health Centre & Canadian Centre for Vaccinology

[5980 University Ave](https://maps.google.com/?q=5980+University+Ave+4th+Floor&entry=gmail&source=g), [4th Floor](https://maps.google.com/?q=5980+University+Ave+4th+Floor&entry=gmail&source=g), R4020, Halifax, NS. B3K 6R8

[akelvin@dal.ca](mailto:akelvin@dal.ca)

VIDO-InterVac

Saskatoon, SK. S7N 5E3

[alk308@usask.ca](mailto:alk308@usask.ca)

(306)-966-3304

[www.akelvinlab.com](http://www.akelvinlab.com/)

**Supplementary Figure Captions and Legends**

**Fig. S1: Uninfected ferrets have minimal change in temperature and weight over 7 days of observation.**

**a** The temperature of ferrets was recorded for 7 days and represented as percentage of original temperature and **b** raw temperature. **c** Weight was also measured for 7 days and is represented as a percentage of original weight. Results show the mean of at least 3 ferrets per group. Error bars indicate +/- standard deviation (SD).

**Fig. S2: Minimal differences of viral RNA distribution in age-matched and sex-matched ferrets.**

Female (1-year-old), male (1-year-old), and male (2-year-old) ferrets were inoculated intranasally with 10^6^ TCID_50_ of SARS-CoV-2 and removed from the study on day 2 and 7 post infection for viral RNA quantification in tissues by qRT-PCR. Viral RNA distribution throughout tissues was compared in **a** females versus males and in **b** 1-year-old males versus 2-year-old males. Salivary gland (SG), trachea (T), right cranial lung (RCrL), right middle lung (RML), right caudal lung (RCaL), left cranial lung (LCrL), left caudal lung (LCaL), accessory lung (AL), mediastinal lymph node (MLN), heart (H), kidney (K), liver (L), spleen (S), and large intestine (LI) were analyzed. The presence of the viral RNA of SARS-CoV-2 was assessed with the Qiagen Quanti-Fast RT probe master mix and primer/probe sets specific for SARS-CoV-2 E gene. An equivalent TCID50/mL was calculated based on CT values corresponding to a standard curve with virus of known titer. Error bars represent standard deviation (SD). Three animals per group were analyzed for both groups. * represent a significant difference between male and female groups as determine by T-test.

**Fig. S3: Gene expression analysis by qRT-PCR indicated 2-year-old ferrets**

**have delayed CXCL10 in lung lobes after SARS-CoV- 2 infection.**

**a** qRT-PCR was performed on RNA extracted from lung lobes of female and male ferrets. **b** qRT-PCR was performed on RNA extracted from lung lobes of 1-year-old and 2-year-old ferrets. The right cranial lung (RCrL), right middle lung (RML), right caudal lung (RCaL), left cranial lung (LCrL), left caudal lung (LCaL), and accessory lung (AL) were harvested on days 2, 7 and 14 post inoculation. Samples were assessed for CXCL10 and IL-6 with specific ferret primers (**Table 1**). Fold change was calculated via 𝛥𝛥Ct against controls with BACT as the housekeeping gene. Error bars represent SD. Three ferrets per group were used for the analysis of all time points. * represents a significant difference as determined by Student’s t-test comparing groups.

**Fig. S4: SARS-CoV-2 infected 1-year-old female ferrets have early upregulation of interferon responses in the nasal turbinates while 2-year-old male ferrets have increased olfactory-associated genes at later time points as determined by RNAseq analysis.**

RNA extracted from infected female and 2-year-old nasal turbinates were subjected to RNA sequencing on the Illumina platform to determine the host transcriptomic responses during SARS-CoV-2 infection. Hierarchical clustering of the Top 50 differentially expressed genes are represented from day 2, 7, 14, and 21 post inoculation. The intensities represent Z-scores from -3 (blue) to +3 (red) in the heat maps. The heatmaps were created by pheatmap (Pretty Heatmaps).

**Fig. S5: Host response profiling analysis by RNAseq of the upper lung lobe (right cranial lung) in SARS-CoV-2 infected ferrets indicated that 1-year-old female ferrets have early activation of interferon stimulated genes.**

RNA extracted from infected female and 2-year-old right cranial lung were subjected to RNA sequencing on the Illumina platform to determine the host transcriptomic responses during SARS-CoV-2 infection. Hierarchical clustering of the Top 50 differentially expressed genes are represented from day 2, 7, 14, and 21 post inoculation. The intensities represent Z-scores from -3 (blue) to +3 (red) in the heat maps. The heatmaps were created by pheatmap (Pretty Heatmaps).

**Fig. S6: Host response profiling by RNAseq analysis of the lower lung lobe (right caudal lung) in SARS-CoV-2 infected ferrets indicated that female ferrets have early activation of**

**interferon stimulated.**

RNA extracted from infected female and 2-year-old right caudal lung were subjected to RNA sequencing on the Illumina platform to determine the host transcriptomic responses during SARS-CoV-2 infection. Hierarchical clustering of the Top 50 differentially expressed genes are represented from day 2, 7, 14, and 21 post inoculation. The intensities represent Z-scores from -3 (blue) to +3 (red) in the heat maps. The heatmaps were created by pheatmap (Pretty Heatmaps).

**References**

**Acknowledgments**

A. Kelvin is funded by the Canadian 2019 Novel Coronavirus (COVID-19) Rapid Research Funding initiative the Canadian Institutes of Health Research (CIHR) (grant numbers OV5-170349, VRI-172779, and OV2 – 170357) and Atlantic Genome/Genome Canada, Scotiabank COVID-19 IMPACT grant, and the Nova Scotia COVID-19 Health Research Coalition. D. Falzarano is funded by the Canadian Institutes of Health Research (CIHR), grant number OV5-170349. M. Cameron, C. Cameron, and B. Richardson are supported by NIH/NIAID (3R01AI129709-03S1) and the Nord Family Foundation, Amherst, Ohio. R. D. Pechous is supported by NIH/NIAID (award R56AI153252). J. Kindrachuk is funded by a Tier 2 Canada Research Chair in the Molecular Pathogenesis of Emerging and Re-Emerging Viruses provided by the Canadian Institutes of Health Research (Grant no. 950-231498). C. Richardson is funded by a CIHR COVID-19 Rapid Funding Opportunity VR1-172779. The authors would like to thank the tremendous efforts of the VIDO-InterVac veterinary staff including Dr. Colette Wheler. We also thank the staff of the CWRU Applied Functional Genomics Core for performing the RNA-Sequencing assays. Operational support at VIDO-InterVac is provided in part by the Canadian Foundation for Innovation through the Major Science Initiates Fund and by Innovation Saskatchewan.

This article is published with the permission of the Director of VIDO-InterVac.

**Author Information**

Contributions

Conceptualization: A. A. Kelvin; Investigation: A. A. Kelvin, M. E. Francis, J. Lew, D. Falzarano, U. Goncin, S. Machtaler; Analysis: A. A. Kelvin, M. E. Francis, B. Richardson, R. D. Pechous, J. Kindrachuk; C. M. Cameron, M. J. Cameron, C. Richardson, M. Rioux; Writing: A. A. Kelvin, M. McNeil, M. Rioux, M. Foley, A. Ge, V. Gerdts

**Ethics declarations**

Authors declare no competing interests.
