# Supplementary figures and images for: Sex and age bias viral burden and interferon responses during SARS-CoV-2 infection in ferrets
Source: Sci Rep. 2021 Jul 15;11:14536. doi: 10.1038/s41598-021-93855-9 (PMC8282673; doi:10.1038/s41598-021-93855-9)

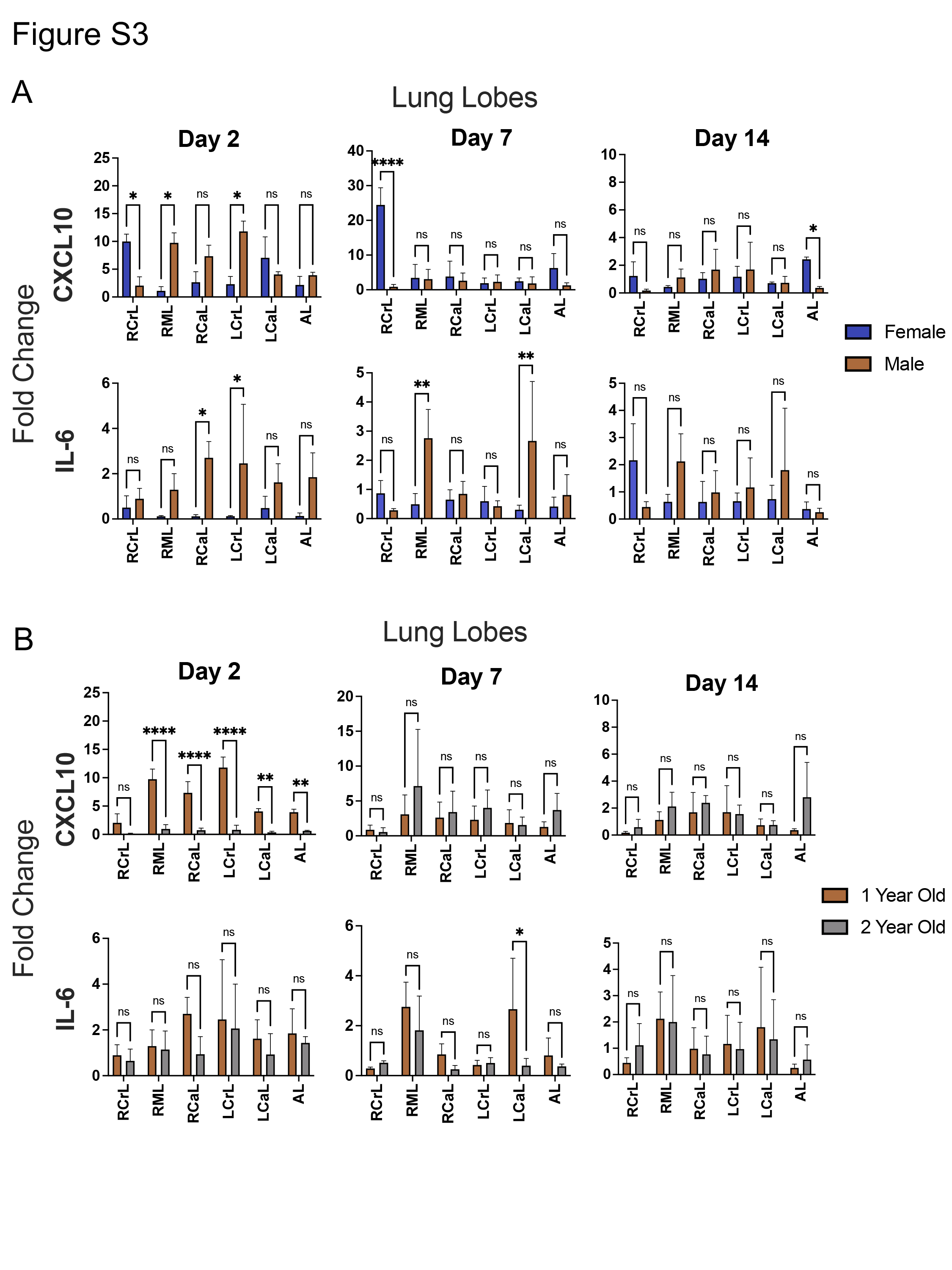

Supplement: Supplementary file 4 — Supplementary Figure S3. [file 41598_2021_93855_MOESM4_ESM.tif]

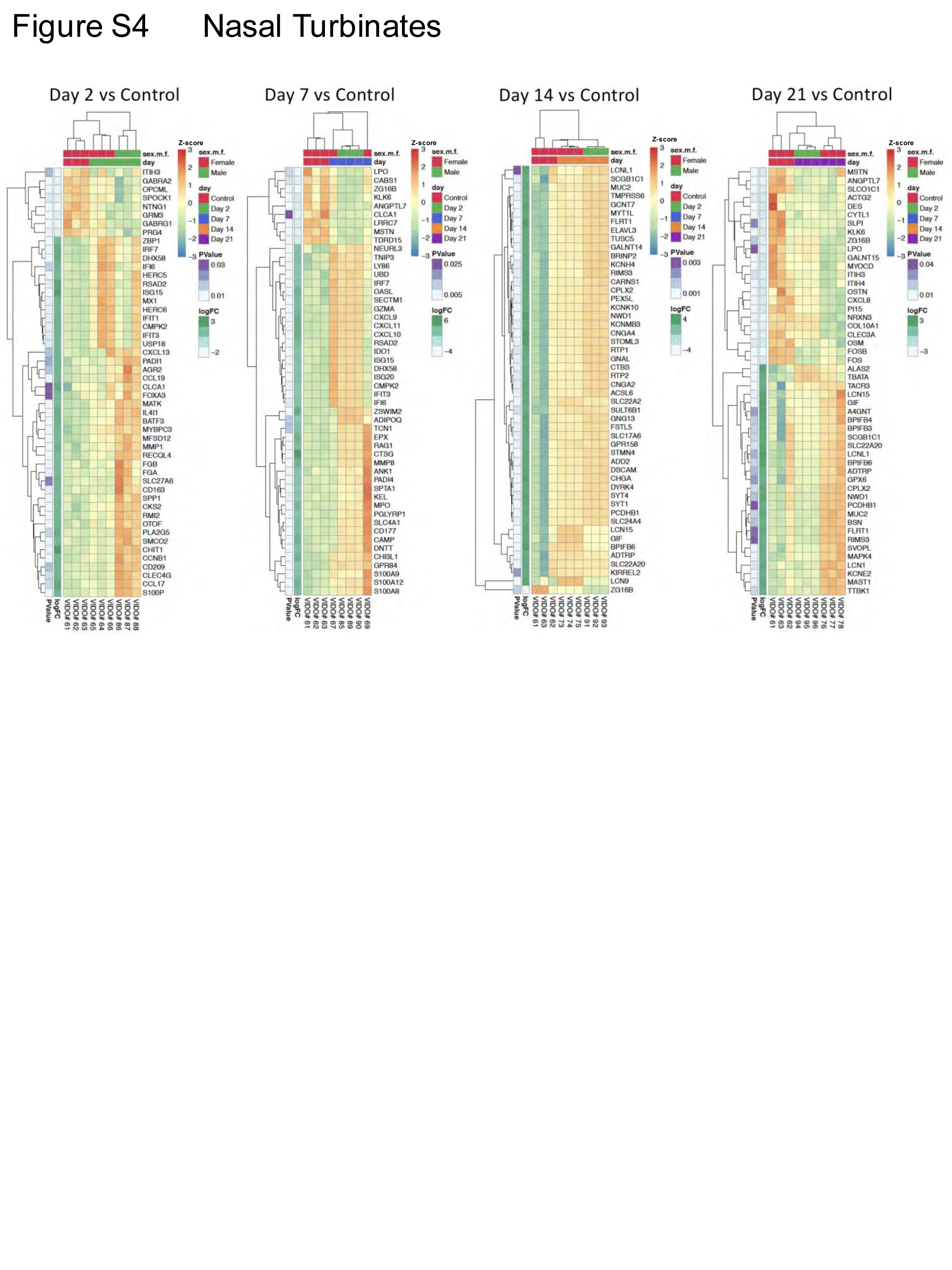

Supplement: Supplementary file 5 — Supplementary Figure S4. [file 41598_2021_93855_MOESM5_ESM.tif]

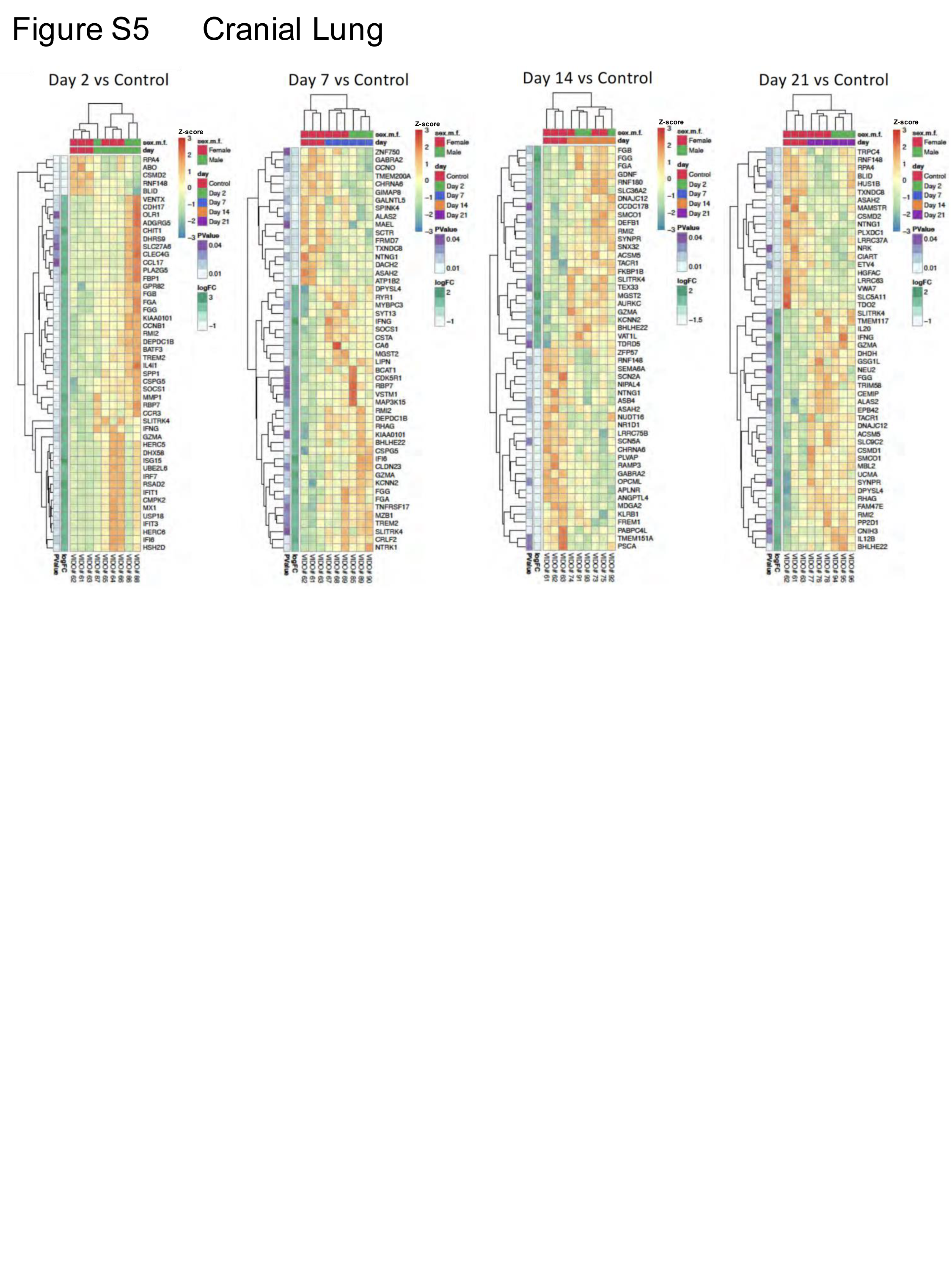

Supplement: Supplementary file 6 — Supplementary Figure S5. [file 41598_2021_93855_MOESM6_ESM.tif]

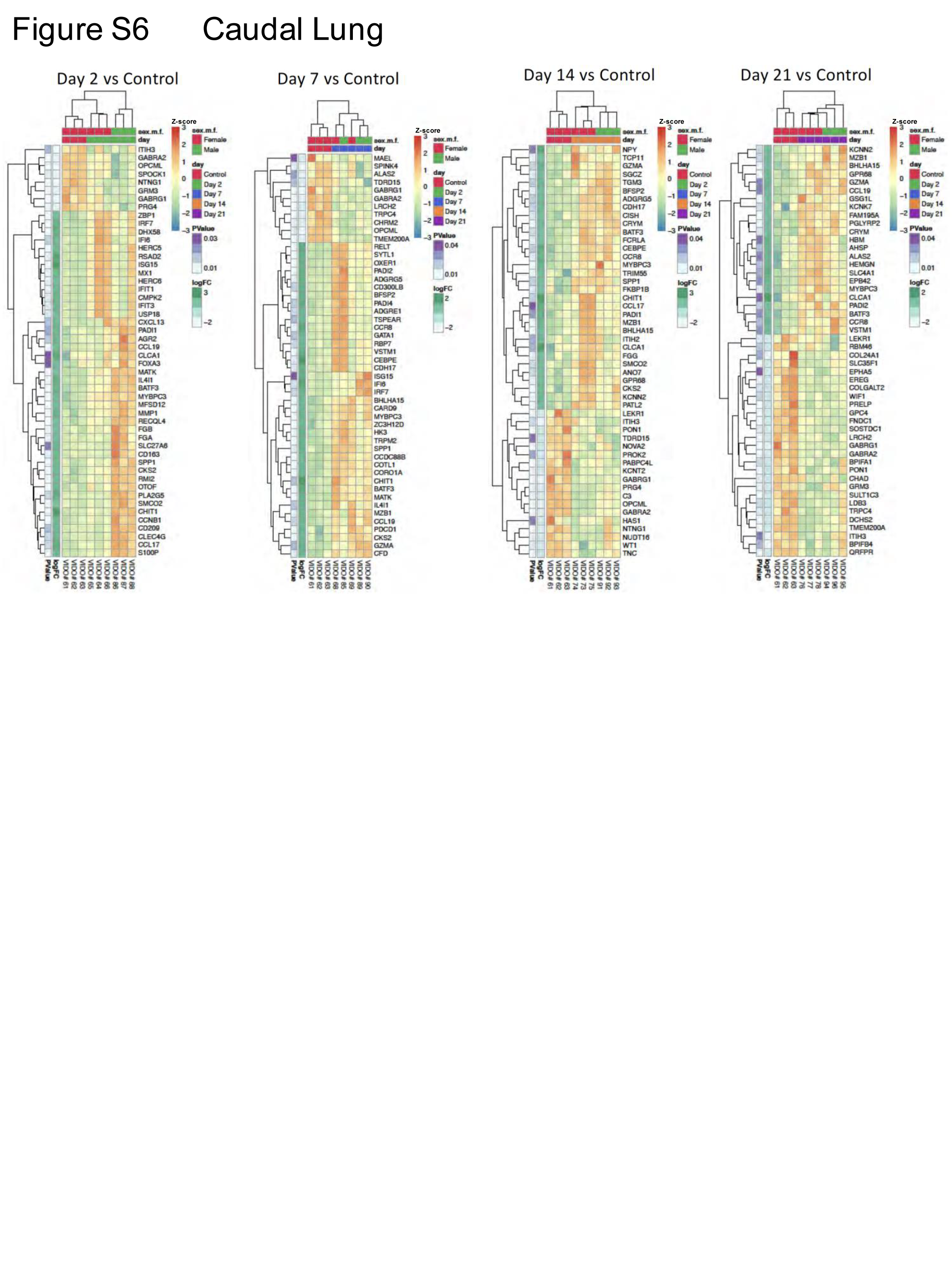

Supplement: Supplementary file 7 — Supplementary Figure S6. [file 41598_2021_93855_MOESM7_ESM.tif]
